# XDH and XO Research and Drug Discovery—Personal History

**DOI:** 10.3390/molecules28114440

**Published:** 2023-05-30

**Authors:** Takeshi Nishino

**Affiliations:** NESA LLC, Yamatomura Greenhouse 501, Honkomagome 6-13-6, Bunkyo-ku, Tokyo 113-0021, Japan; takeshinishin@gmail.com; Tel.: +81-3-3947-1150

**Keywords:** xanthine dehydrogenase, xanthine oxidase, molybdopterin, non-heme iron, flavine, uric acid, gout, purine metabolism, reactive oxygen, COVID-19

## Abstract

The author will outline the research history of the main issues addressed in this paper. The author has worked on this research himself. XDH, which is responsible for purine degradation, is present in various organisms. However, conversion to XO only occurs in mammals. The molecular mechanism of this conversion was elucidated in this study. The physiological and pathological significance of this conversion is presented. Finally, enzyme inhibitors were successfully developed, two of which are used as therapeutic agents for gout. Their wide application potential is also discussed.

## 1. Introduction

**Figure 1:**
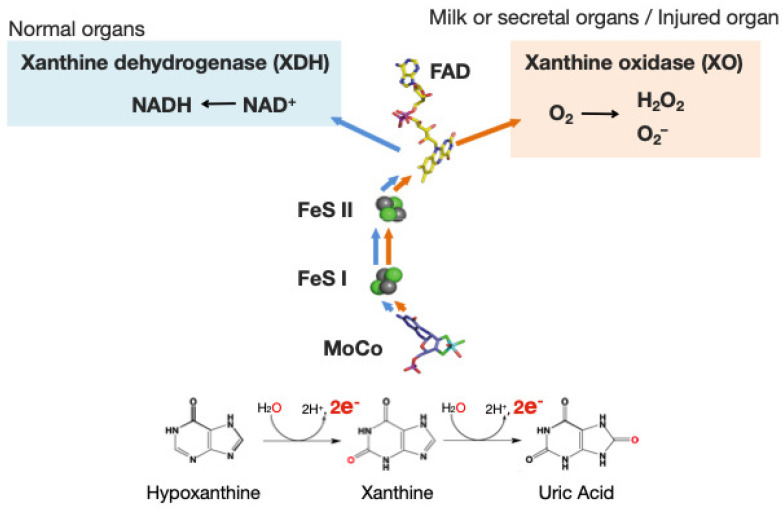
Illustration of XOR reaction from the X-ray structure.

This enzyme, XDH, was extracted from cow’s milk in 1902 [1], more than 120 years after its discovery now. It was considered one of the most difficult enzymes to study when I graduated from medical school. There is even an anecdote that when the genius Warburg first started researching this enzyme, he gave up after two weeks, saying it was a ‘bad enzyme’. It was then purified by Ball E.G., a pupil of Warburg for a short time, and in 1939, he published a paper showing that flavin was one of its constituent components [2]. Until approximately the beginning of 1970, the composition and history of the progress of this enzyme, including the Mo-containing enzyme discovered by RC Bray, and its properties were comprehensively described in more detail [3]. It is a dimeric enzyme with a total molecular weight of about 300 kDa, and each subunit contains Mo metal, two non-heme iron [2Fe2S] molecules, and FAD as cofactors. However, obtaining reproducibility in every experiment was generally known to be impossible. That was a challenge in researching this enzyme. In the 1960s, RC Bray and V Massey et al. revived the study of this XO enzyme from bovine milk using rapid EPR and stop-flow methods combined with absorption spectroscopy, respectively, to study the enzyme and its properties [3,4,5]. In their study, the Mo metal, two non-heme iron [2Fe2S] molecules, and FAD present were studied in detail, and the properties of the enzyme itself were well advanced. It was impossible to reproduce every experiment because the Mo in the enzyme contained an inactive form and had a variable content [6]. Furthermore, Massey and his co-workers found that the reactivity of the coenzyme, due to the distribution of electrons to each redox center in response to each redox potential, was ultimately explained by its reactivity with oxygen [4,5]. In the meantime, Stirpe F and Della Corte E reported something surprising: the mammalian XO was originally an XDH type changed to XO by cleavage by proteolysis at the specific site of the enzyme protein or the formation of S-S bonds [7]. The difference between XDH and XO is whether the enzyme receives two electrons from hypoxanthine or xanthine and passes them to O_2_ or NAD, as illustrated in Figure 1. At this time, I began to study the XDH enzyme in chickens that never converts to XO. I have been studying this enzyme XDH and XO for nearly 50 years since then. The study provided an improvement in the purification of the enzyme, as shown below. Since then, I have studied the enzyme itself in relation to the mechanism of conversion of XDH to XO, the related purine metabolism, and drug discovery. It is difficult to describe its various aspects. Here, I will only briefly describe the important origins of my research, the main results, and the background and progress of my research. Finally, why mammals need XDH to change to XO is of great interest and will be discussed in this paper. The author’s references in this description are therefore limited, and many of the important papers also written by other researchers can be found in the references of the respective articles.

Here, the reaction shown in the figure represents the degradation of purines. The degradation reaction is oxidized by MoCo, where either hypoxanthine or xanthine is oxidized, each with two electrons being transferred to Mo through two Fe/S molecules in the enzyme to FAD, two electrons are transferred to NAD in XDH, and two electrons are transferred to O_2_ in XO. In general, naming enzymes is a commitment to naming those that pass to O_2_ as oxidases and all others as XDH. This is why the same gene product is sometimes called an XOR enzyme, as the enzyme can be either XDH or XO, depending on the state.

## 2. Origin of My Enzyme Research

**Figure 2:**
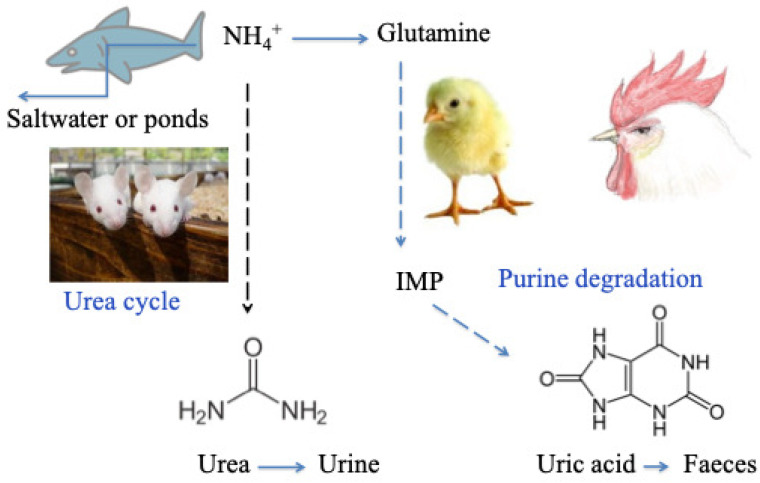
Illustration of the final processing of the amino group of amino acids.

**Figure 3:**
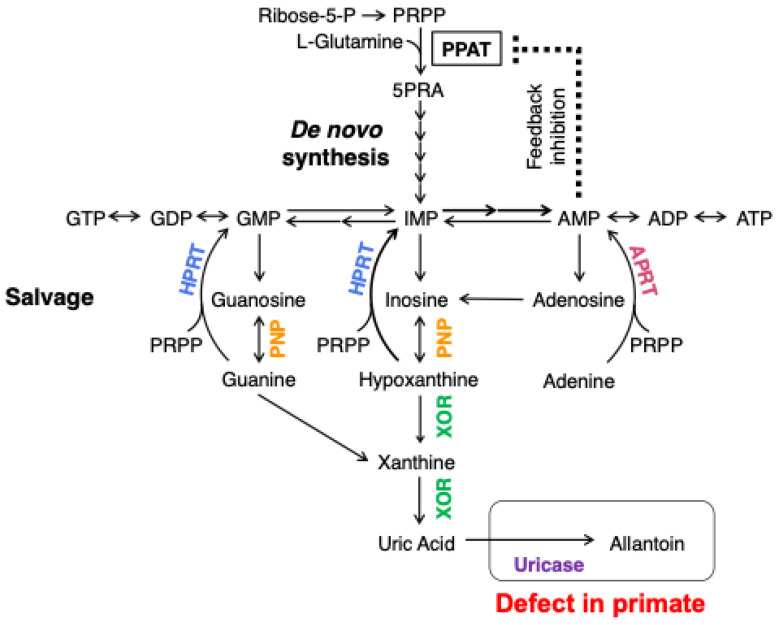
The metabolic pathway of purine metabolism.

I was born in Tokyo in 1944. It was during the war, and soon after my birth, Tokyo was exposed to an air raid. In this air raid, incendiary bombs were dropped around the city, blocking the way for the city’s residents to retreat. The indiscriminate bombing was carried out in the inland areas, resulting in many deaths. The indiscriminate bombing resulted in the massacre of civilians. In countries with no democracy, the victims of war are always the weakest and the most unaccountable. It was a miracle that I survived. This was because my family had been evacuated to a remote village just before the bombing. Living in the village gave me a life experience I did not have in Tokyo. I kept chickens at home and nourished them with eggs. This poultry farming experience, fortunately, helped me in my research at university. In those days of postgraduate life in medical school, there was very little money for research in the laboratory. No real grant system in Japan at that time allowed many researchers to apply. In this environment, I was fortunate. I obtained male chickens from a poultry farm. The males, which did not lay eggs, were obtained free of charge and for a labor charge only. Chickens are uric-acid-producing animals. The amino acid nitrogen is excreted as uric acid rather than urea, as occurs in mammals (Figure 2). Purine-degrading enzymes, as shown in the purine metabolic pathway (Figure 3), are considerably more active in chickens than in mammals. It is even higher in diets high in protein as a nitrogen source. The activity of xanthine dehydrogenase, the enzyme of uric acid conversion, has been found to be higher when the protein content of a diet is high (activity per FAD) [8]. It has also been found that this is due to the amount of S atoms present in the MoCo, which is essential for the enzyme’s activity, it being present in samples of the enzyme [8]. The number of S atoms changes with each purification because the S atoms are unstable (they are easily replaced by O atoms during storage or during CN ion treatment). These were major obstacles to experiments on this enzyme in terms of obtaining reproducible results [9,10]. The difference between flying birds (pigeons) and non-flying chickens is also interesting. In chickens, XDH acts in the liver, whereas in pigeons, XDH is only present in the kidneys [11]. This is considered to be likely an evolutionary difference between flying birds, which avoid water loss by excreting solid uric acid in the kidneys, and chickens, which walk on the ground without flying.

Amino acids are transferred to glutamate by transaminases, and the nitrogen produced by the oxidative deamination reaction needs to be processed. In fish, this becomes ammonia, which is discharged directly into seawater, lakes, and rivers for disposal. In mammals, on the other hand, it becomes urea in the urea cycle and is disposed of as a component of urine. Birds, conversely, synthesize purines, which are eventually converted to uric acid and disposed of as solid feces. Birds generally have a high XDH activity, which is even higher if they are on a high-protein diet.

Here, the reactions of purine synthesis and its degradation are represented; the schematic diagram shows the degradation of purine and the reaction of HPRT to recover hypoxanthine.

## 3. Establishment of a Fully Active Enzyme: Purification Method from Bovine Milk

The method of purifying this enzyme was devised to separate S-atom-deficient (replaced by oxygen (O) atoms) enzymes from enzymes with almost fully active enzymes [12]. The S atom is rather stable in the reduced state but is removed by CN [13]. The invention and establishment of this method took five years; it was carried out by visiting the laboratories of V. Massey and R.C. Bray. Both observed with amazement and acknowledged the enzyme’s high activity and the method’s correctness. The preparation of the column, its use, and the activity of the original enzyme are relevant. Without an accurate understanding of the principles and methods, it is impossible to carry out each step of the method [14]. It is impossible to obtain a fully active form of the enzyme from milk with a low XO activity (because there is no fully active form there). Therefore, the cow used is important for obtaining the highly active form of the enzyme. This is because the cows’ feed will determine how much of the highly active form is in the milk. This was learned from the case of chickens, as discussed above.

It has also been reported that the lower the molybdenum content in cattle diets, the lower the XOR activity of milk [3]. Purified enzymes must not be stored frozen. The enzyme should be stored on ice in a cool, dark place together with a small amount of salicylic acid; the salicylic acid is removed with G-10 immediately before the experiment, and the experiment is completed within 12 h while stored on ice in a cool, dark place (normally covered by black cloth). This is a very cautious experiment. Furthermore, in the cDNA expression system described below, the molecules produced using the gene expression method are diverse macromolecules with multiple complementary molecular species [15]. This is due to the complexity and nature of the huge enzyme, which has multiple cofactors, and His-Tag can only be used for simple enzymes. Despite patience and effort, it was initially possible to collect molecules by using molecular mass columns of around 300 kDa. Thus, it is not so troublesome. The active enzyme can then be obtained by folate affinity chromatography as described above. The valuable experience gained in that study was used in all subsequent studies. It is gratifying that the laboratories of V. Massey, RC Bray, Russ Hille, and others have taken over this method, implemented these methods, and developed the chemical results, such as the reaction mechanism of this enzyme. For example, Russ Hille and Takeshi Nishino took over and implemented these methods and published their results, including the reaction mechanisms and kinetic data [16].

## 4. The Method of Extraction and Purification of XDH from Rat Liver

XDH (xanthine oxidoreductase) enzyme is easily converted from XDH to XO as described above. We tried to isolate the only XDH form in rat liver. The enzyme exists in a cytosol fraction [17]. This requires careful homogenization that avoids destroying organelles to ensure that lysosomes are destroyed during homogenization so that irreversible conversion by proteases does not occur. To this end, using a high mitochondrial P/O ratio as an indicator was important. Doing so gently uses a loose Teflon glass homogenizer and avoids negative pressure inside the vessel [18,19]. Like with any good dish, it requires repeated practice. Based on these results, the author collaborated with V. Massey, who had been interested in this enzyme for many years and had analyzed its properties from various angles, especially using the stop-flow method; V. Massey became a visiting professor at Yokohama City University at the time I was an associate professor and carried out a lot of research to clarify the properties of the enzyme. He achieved several results in clarifying the nature of enzymes. The usual practice is to use pure enzymes to obtain correct results. He and our group obtained many results in a short time [20,21,22,23,24,25,26].

## 5. CDNA Cloning and Determination of Primary Structure from Various Sources

Fortunately, with the development of the Japanese economy, a grant system was established, and it became possible to apply for and obtain research funding. The development of the grant system made it possible to obtain considerable research funding and to analyze the primary structure and reaction mechanism of this enzyme by cloning cDNA in rats [27], chickens [28], and humans [29]. In other words, elucidating the crystal structures of xanthine dehydrogenases and oxidases and the various reaction mechanisms in more detail would have been impossible without it. The methods used back then involved much more time than they do today, as sequencing was done by hand. The difference is that it takes a few minutes now where it took more than two years in those days.

## 6. Determination of the Crystal Structures and Site-Directed Mutagenesis Studies

**Figure 4:**
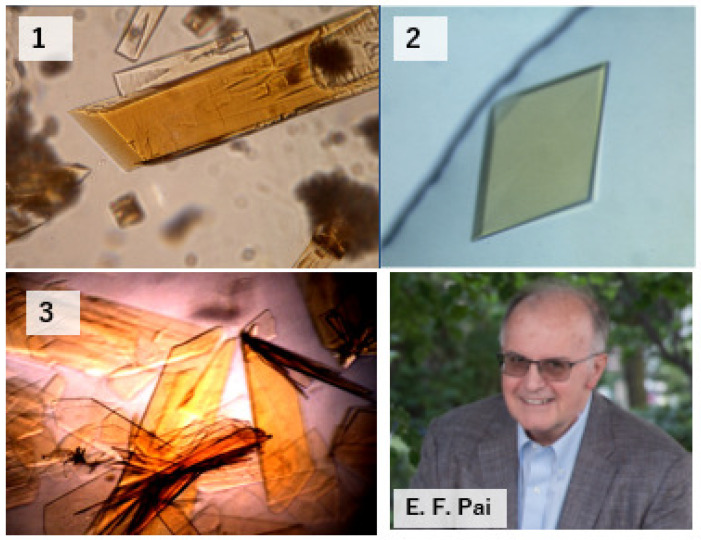
Micrographs of bovine milk crystals. XO was obtained in 1986 (1), in 1998 (2), and XDH was grown in 1998 (3), and Emil F. Pai in bottom-right panel.

Fortunately, while I was working in V. Massey’s laboratory, the crystallographer Emil F. Pai (Figure 4), who was then at the Max Plank Institut für Medizinische Forschung in Heidelberg, visited Massey’s laboratory and developed an interest in the analysis of the crystal structure of XO. He quickly learned that he could not crystallize the enzyme extracted from milk by butanol treatment. After returning to Japan, I produced the crystal using the enzyme extracted from cow’s milk by the pancreatin treatment method. I then visited Heidelberg myself and handed over the crystals. However, the analysis was not easy. He told me that all the pancreatin-treated crystals were twins, i.e., two crystal lattices penetrated each other while being offset by about a 1° angle. While Pai’s experience had shown that crystals could not be obtained from protein obtained by butanol extraction, crystals made from pancreatin-treated protein did not lend themselves to successful analysis either. It took me ten years to realize the reasons behind this. We presumed that, for one, the mixture of fat inhibited crystallization, and the cleavage of proteins and peptides was disadvantageous. I thought that a special process was needed to remove lipids. I therefore arrived at the idea of lipase treatment combined with inhibitors of proteolytic enzymes [30]. As a result, a crystal structure was successfully obtained, first of XDH and then also of XO in 2000 [31]. Pai’s efforts to elucidate the crystal structure of this difficult enzyme were not in vain. We succeeded in analyzing this enzyme’s reaction mechanism, especially the substrate’s binding mode [32] and then the reaction intermediates [33]. The structure of this reaction’s intermediates was found to be reversible and to be exactly the reaction intermediates. It had been previously discussed by Bray et al. that this enzyme accepts a rather wide variety of compounds as substrates. It reacts not only with xanthine but also with hypoxanthine and even allopurinol. Furthermore, it can be seen from its history that it was originally found as an aldehyde oxidase. It is, therefore, important to understand how the substrates bind with it and the rate at which the product dissociates from it, which are also influenced by the state of the surrounding amino acids. Unsurprisingly, the discussion cannot be based solely on slight differences in activation energies. It is also important to consider the influence of the oxidation state of each cofactor mentioned in the introduction. A paper dealing with these aspects will be published in the near future. The mode of binding is rather widely inferred from the reaction mode of MoCo. Furthermore, crystallographic analysis has provided a detailed understanding of the reversible and irreversible changes from XDH to XO that the enzyme undergoes [34,35]. As mentioned, purifying XOR enzymes from living organisms is very difficult. On the other hand, to analyze the conversion mechanism of XDH to XO, it was necessary to mutate each amino acid in the enzyme molecule by gene expression methods and compare the crystal structure with the active conversion from XDH to XO. In this case, insect cells were used because introducing a complementary family of molecules in the *E. coli* bacterial expression system could not keep up with the sufficient production of MoCo and [Fe_2_S_2_]. Various defective XOR species were produced even with insect cells, but this isolation was relatively easy compared to rat liver cells. When the paper on this expression system and purification of XDH or XO was submitted to the JBC journal, it was immediately rejected. The editor described it as too complex and ‘unusable’. However, from my point of view, it was much easier than purification from liver, which required a great deal of effort. Anyway, using “this unavailable method”, we analyzed various mutant amino acids. Using this experimental system, the mechanism of the conversion of XDH to XO and the conversion of the protein molecule was greatly advanced [36,37,38,39,40]. 

XDH crystals are an excellent example of the idea that “inner values”, e.g., crystalline order, are more important than “outside appearance”, e.g., clean shapes, at least when it comes to protein crystals.

**Figure 5:**
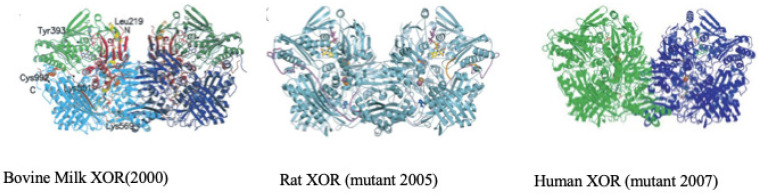
Illustration obtained from various types of XDH or XO material.

**Figure 6:**
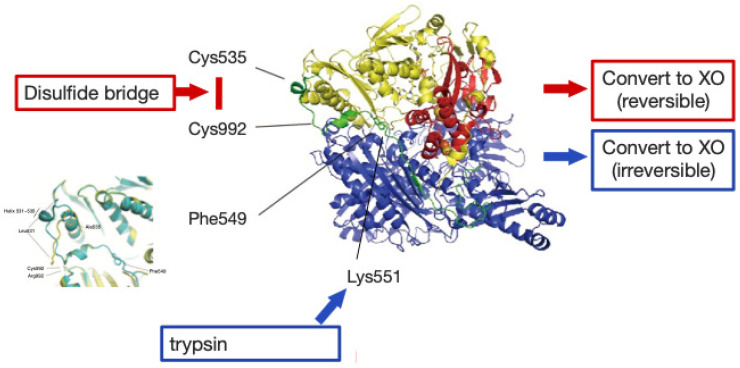
Illustration of the mechanism of conversion from XDH to XO.

The overall crystal structure of the XOR enzyme is shown in Figure 5. The overall images are shown to be similar. The details vary greatly between XDH and XO. The text gives an overview and background of the research into the analysis of the conversions. Using this experimental system, the mechanism of the conversion of XDH to XO and the conversion of protein molecules was greatly advanced [36,37,38,39,40]. The conversion of XDH to XO (Figure 6) and the conversion of protein molecules were carried out simultaneously to elucidate their various crystal structures. 

The structural sites responsible for the change from XDH to XO are briefly summarized here.

## 7. Development of Enzyme Inhibitors and Their Analysis as Drugs

In developing XOR inhibitors for gout treatment, even when testing different enzyme inhibitors (strength and mechanism of inhibition), the starting point of enzyme research—‘getting the right result with a good enzyme’—was essential. Highly active enzymes and analytical methods were essential, especially in strength testing in the case of XO. At that time, commercially available enzymes had less than 1% activity. It must also be noted that there have been no successful attempts to select its efficacy as a gout drug by oral administration in animals such as mice. This is because the activity of hypoxanthine phosphoribosyl transferase (HPRT) is significantly higher in humans than in mice (manuscript in preparation).

Therefore, highly active enzymes and analytical methods were also essential for this strength test. Gout is a disease in which large amounts of uric acid accumulate and crystalize, causing inflammation. Initially, researchers from Otsuka Pharmaceutical Co. visited the laboratory in around 1985 and a researcher from Teijin (S. Kondo) visited the laboratory in 1988. A researcher from Fiji Yakuhin Co. (K. Matsumoto) then visited the laboratory in 2002, followed shortly afterward by a researcher from Mitsubishi Pharm. Co. (A. Fukunari). Finally, two drugs were successfully developed and marketed.

Teijin’s Kondo finally succeeded in obtaining a good inhibitor (Febuxostat), but he did not have an accurate and in-depth understanding of allopurinol’s true mechanism or strength. He stated that “it has an IC_50_ lower than allopurinol”. In this respect, he was similar to those who misunderstood allopurinol’s true mechanism and strength and failed to develop inhibitors. Fortunately, I understood the mechanism of allopurinol through enzymatic purification, as mentioned above. I told him that allopurinol is a suicide substrate and is very strong but that the binding has a half-life and does not last long, which is a weakness of allopurinol. My experience with allopurinol in the purification of enzymes came in handy here. The first inhibitor Kondo brought was weak, so I told him that the nano level of Ki for xanthine was essential. The next enzyme inhibitor he brought was indeed less than nano Ki, but I told him not to use it because it contained a carcinogen called NO_2_, which is dangerous. This fact was also pointed out experimentally in the carcinogenicity research department of Teijin. Thus, he changed the substance he brought with him to one with a nitrile group and measured its activity, which turned out to be very strong. Kondo thought the mechanism of this strength was that the pterin part of the inhibitor paralleled the pterin ring of MoCo and stopped the electron transfer reaction. I did not think so because any indication of a small charge transfer was not observed in absorption spectrum experiments. These data showed a mixed-type inhibition, as did the BOF analyzed earlier [41]. The mechanism was almost identical, and the strength was different. I told him not to publish the data as they needed to be clarified more, and I handed them over to him. In fact, subsequent X-ray crystallographic analysis revealed that the nitrile groups interact ionically with the protein’s amino acids, enhancing the inhibitor’s binding. We believe our findings will significantly contribute to the basic research on the XOR enzyme. I carried this out because I was used to measuring and analyzing the enzyme activity of stronger inhibitors. With stronger inhibitors, using the usual electron acceptor kinetics is difficult, and it is necessary to change the measurement method between the physiological form, XDH, and the converted form, XO. Both XDH and XO are active, and the inhibitor competes with xanthine and hypoxanthine at the MoCo site, directly passing electrons from Mo to other compounds. Still, therefore, a method was devised to observe the strength of inhibition by binding PMS and cytochrome c [41]. This method tested XDH and XO enzymes without distinguishing between the two types of enzymes and had a good sensitivity. This method was essential because of the special experimental experience conditions and failures behind the success of Febuxostat. 

**Figure 7:**
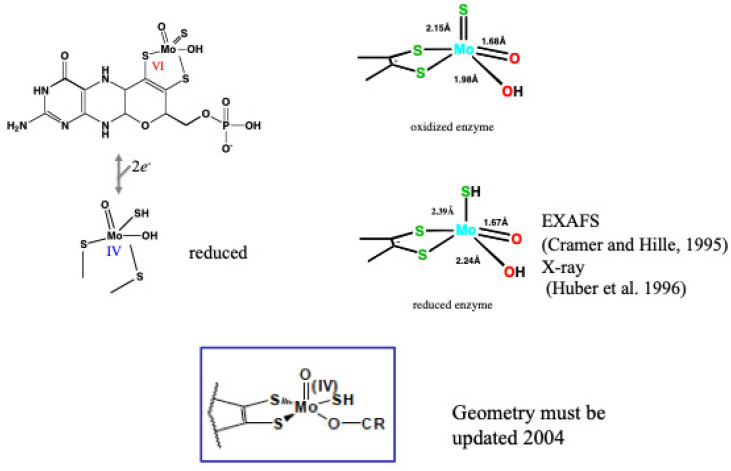
Geometry around Mo atom obtained from X-ray structure during catalysis. The picture was obtained bound with FYX-051 [44].

Illustrations of the structures of the inhibitors at the active centers of XORs were developed in Japan and studied by the authors of this paper, respectively. The mechanism and strength of inhibition are not the same conditions for each; see the results in the following papers: BOF-4272 [41], TEI-6720 (Febuxostat) [42], Y-700 [43], and FYX051 (Topiroxostat) (Figure 7) [44,45] surrounding proteins. The dynamic structure around the MoCo of the bacterial enzyme, which will be discussed below, was also revealed.

Topiroxostat showed that it was not just an inhibitor but also provided important information on the reaction mechanism because it is a reaction intermediate. It showed the role of each amino acid and suggested the reaction mechanism in the purine substrate hydroxylation. It also provided information on the accurate geometrical position of the S atom and others in the active center [44].

Furthermore, the following important roles of the XORs and their associated metabolic kinetics were elucidated. The information provided a better understanding of purine metabolism.

**Figure 8:**
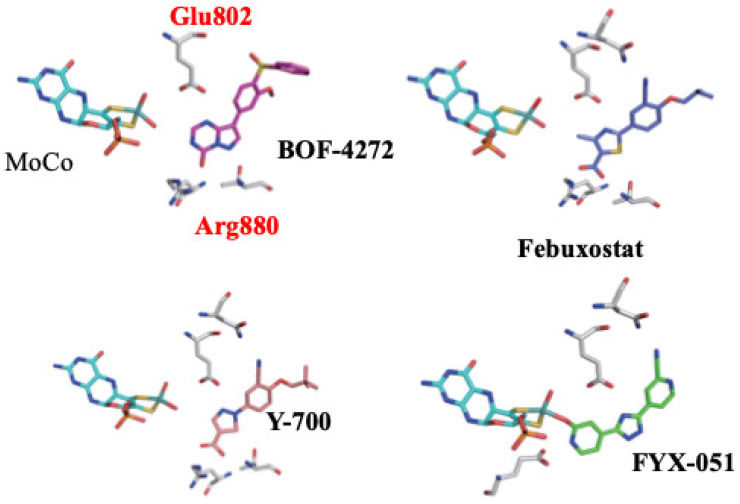
Various XOR inhibitors within the active site of XOR, showing X-ray structures that were analyzed in our laboratory.

XDH or XO catalyzes two steps: the conversions of hypoxanthine to xanthine and xanthine to uric acid. It should be noted that the most important point is the inhibition of the hypoxanthine-to-xanthine step rather than the xanthine-to-uric-acid step. The Km of hypoxanthine is low, so the inhibition needs to be strong enough so that the Ki value is less than a nanomole of the Km for xanthine. This means that, in long-term administration, the effect of XOR inhibitors is to inhibit the conversion of hypoxanthine to xanthine, with the increased hypoxanthine being rescued by hypoxanthine phosphoribosyl transferase and the resulting increased AMP (via IMP) being able to inhibit the first reaction of purine synthesis. This is because it can allosterically inhibit the first reaction of purine synthesis. There are risks, as it is also associated with increased ATP in various cells, so care should be taken not to start or stop the drug abruptly. On the other hand, a deficiency in the purine salvage circuit is known as the so-called Lesch–Nyhan disease. Defects in this enzyme (HPRT) are known to cause severe neuronal disease and significant brain atrophy. Genetic disorders such as Down’s syndrome show an increased synthesis of purine bases and show hyperuricemia. This indicates that the salvage pathway and purine de novo synthesis are crucial for neurons. In a mouse model of ALS [46], a neurological disease, we renewed the HPRT with an XOR inhibitor and observed a suppression of progression, although HPRT activity in mice is known to be much lower than in humans. Based on these results, we are currently searching for agents with protective effects on neuronal cells to prevent the progress of my starting point of obtaining good results with good enzymes being essential for the testing of various enzyme inhibitors (strength and mechanism of inhibition), namely the development of XOR inhibitors as gout drugs. For this, strength tests were variously studied using pure and active enzymes. Gout is a disease caused by high levels of uric acid that build up, crystallize, and cause inflammation. The success of this drug led to the creation of Febuxostat (Mr. Kondo from Teijin came to us in 1988) and Topiroxostat (Mr. Matsumoto came to us in 2002). As shown in Figure 8, several other agents were promising but somewhat less potent than the above-mentioned two and were not marketed. However, the failure of the drug’s analysis and marketing experience gave the secret to its subsequent success, and a successful company can only be said to be lucky. Febuxostat is the product of the result of the idea to make it less than nano, which was learned from the failure of BOF-4272 of this author [41]. Theoretically, XDH or XO are enzymes that catalyze the two steps from hypoxanthine to xanthine and from xanthine to uric acid. It is important to note that it is not the xanthine-to-uric-acid step but the hypoxanthine-to-xanthine step that is inhibited. Since the Km of hypoxanthine is low, strong inhibition is required such that the Ki value is less than a nanomole of the Km of xanthine. In other words, in long-term administration, the effect of XOR inhibitors is to inhibit the conversion of hypoxanthine to xanthine, to rescue the increased hypoxanthine with HPRTase (hypoxanthine phosphoribosyl transferase), and the resulting increased AMP (via IMP) to allosterically inhibit the primary step of purine synthesis. This is due to the allosteric inhibition of the initial stage of purine synthesis from amino acids. There is no increase in xanthine or xanthine in urine when using this method. This is the same logic as in XOR deficiency.

## 8. Bacterial XDH

**Figure 9:**
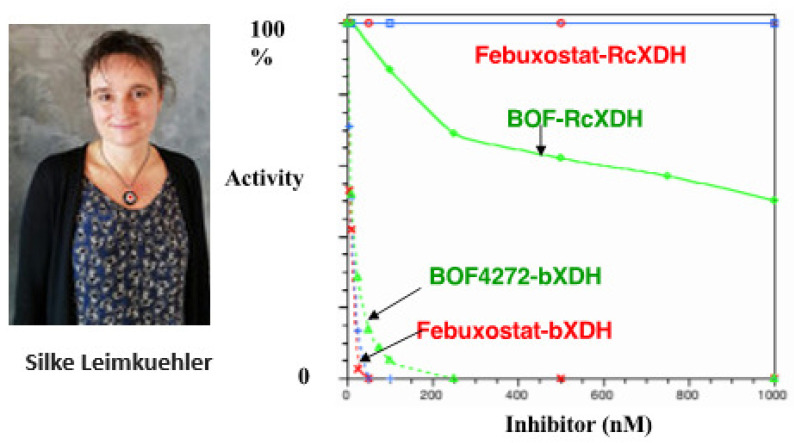
Behavior of two inhibitors of bacterial XDH.

Another important aspect of XDH was learned from this bacterial enzyme. Silke Leimukuller, from Germany, was invited to Tokyo. Various inhibitors were ineffective against this bacterial enzyme, except for allopurinol. The steric structure was apparently similar to a molecular replacement that was obtained from milk enzymes. However, its behavior towards inhibitors was very different. The reason for this was not the active center but the kinetic properties of the protein structure of the enzyme. Furthermore, it has already been reported that the enzyme XDH is converted to XO only in this mammalian species. Remarkably, this bacterial enzyme is always stable. The S atom of the active center, MoCo, was very stable and did not alter its activity. The reaction kinetic analysis using the stop-flow method was relatively stable [47]. However, the effects of inhibitors on this enzyme were unusual. Allopurinol inhibited it, but its mode of binding to the protein appeared to be in a different orientation from that of mammals. Surprisingly, as shown in Figure 9, TEI (Febuxostat), which strongly inhibits mammalian XOR, did not inhibit the bacterial enzyme she brought with her at all (100% enzyme activity was preserved). In contrast, BOF, which was an order of magnitude weaker, did inhibit the enzyme to some extent (Figure 9). This was related interestingly to fluctuations in the surrounding proteins [48]. Therefore, it was also found that enzymes from non-mammalian animals were not directly applicable to enzymological, physiological, pathological, or pharmacological studies.

When two inhibitors, Febuxostat, which is strong against the mammalian enzyme, and BOF, which is one order of magnitude weaker, were added to the bacterial enzyme to see their inhibitory effects, Febuxostat did not inhibit it at all. At the same time, BOF was weakly inhibitory [48].

## 9. Other Medical Aspects

I had graduated from medical school and therefore had some medical knowledge at the time of conducting the study. It was a combination of basic biochemistry and medical knowledge. The enzyme’s deficiency was evidenced by the cloning of the S atom of Mo=S incorporated in the enzyme and the discovery of the patient, as well as a presumptive XDH deficiency type II (see below, [49,50]) due to a deficiency of the enzyme itself (type I) [50,51] and the S-atom-containing enzyme required for activity, discovered at the start of the study. Here, the enzyme required for Mo=S was proven for the first time in a patient with type II deficiency [49,50].

Finally, I would like to discuss the physiological significance of the conversion of XDH to XO and the application of enzyme inhibitors to related diseases; even a narrow range of enzymes is highly relevant to various diseases; the XDH → XO reaction has physiological significance due to its unique role in mammals in vivo [52]. As already mentioned, phylogenetically, only mammalian lineages are converted; in all tissue cells below the avian lineage, the enzyme is not converted to XO but only to XDH. The authors believe that in mammals, the conversion from XDH to XO is specific to specialized mammary glands (extremely weak activity in humans) and salivary glands, where the highly reactive reaction intermediate oxo-thiocyanate acts as a defense against bacterial infection during milking. In other words, it is consistent with the innate immunity theory of the ‘mammalian’ route, which is specific to mammals: converting XDH to XO requires XDH itself, LPO (lacto-peroxidase), thiocyanate, and a substrate, hypoxanthine, or xanthine. This conversion mechanism has also been described in SARS. Furthermore, this conversion mechanism may cause SARS-CoV-2 proliferation and inflammation; the S-S bond may serve as a nursery ground for the virus, making it essential. Unsurprisingly, the oxothiocyan produced by converting XDH to XO by the above mechanism contributes to ER stress.

These facts make it highly questionable as to whether XOR inhibitors are effective as antiviral agents at the doses used for gout in COVID-19. Viral infection requires strong action on the specific cells infected. In other words, the concentration must be high enough to prevent the conversion of XDH to XO in infected obliterating cells. In fact, oral administration of this inhibitor has shown very low efficacy in clinical trials. Most XOR inhibitors work in the liver and have very low blood levels, so their effectiveness is marginal, and they are not used as effective drugs to treat the virus. Furthermore, XOR inhibitors, as already mentioned, increase the concentration of hypoxanthine in the blood, which is also a factor in helping the conversion of XDH to XO at the site of infection. Special pharmacokinetic considerations are needed. Furthermore, the efficacy of XOR inhibitors has been evaluated in some cases using kidney slices. Still, such kidney slices may destroy some cells during sectioning, and the protease is irreversibly converted to XO. Furthermore, they may have been exposed to air and converted to XO by S-S bonding. Therefore, testing it as a viral agent is unreliable. Special methods are needed.

**As for reactive oxygen species,** there is the so-called systemic reperfusion injury theory of reactive oxygen species (ROS) after conversion to XO [53]. The theory generated great interest and influence. It was cited an enormous number of times. However, after a dispassionate review of the theory, and after looking for and discussing the results of corresponding positive and negative papers, it was concluded that the theory was inconsistent [54]. We compared two groups of mice, in which the XOR gene in the mice was replaced by an enzyme gene that does not change to XO and a mutant gene that produces large amounts of superoxide, respectively. Both groups were unchanged during growth and death; no symptomatic differences were observed. Therefore, together with the above discussion on humans, we considered this theory to be incorrect. The so-called systemic reperfusion injury theory of ROS is not likely to be true; it did not affect the growth and lifespan of mice that release high amounts of ROS [55]. This also contradicts numerous papers’ theories that uric acid prevents dementia in the brain by removing ROS. Indeed, uric acid is barely detectable in the human brain, and XOR is not detected. Nevertheless, a forthcoming paper will present the reasons why purine metabolism is important for neurons.

## 10. Collaboration with Researchers at Home and Abroad

**Figure 10:**
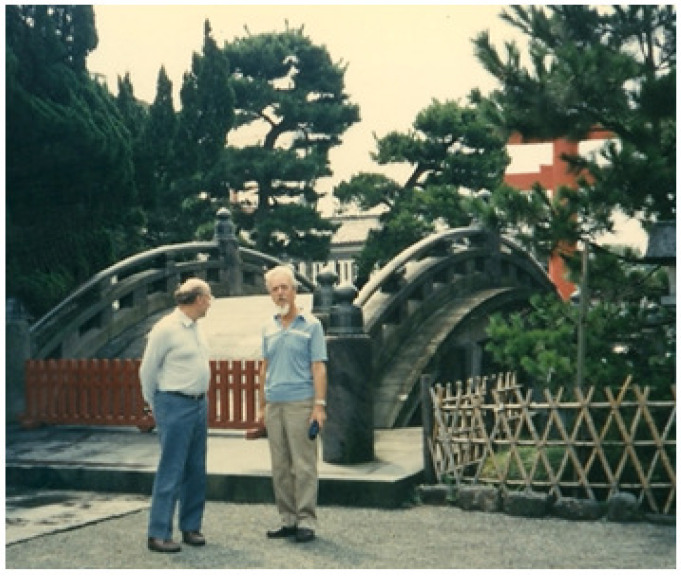
This photograph was once taken when Vince Massey and Bob Bray happened to stay at our house for a week and visited Kamakura one day in between. Unfortunately, however, they have both passed away. They both greatly influenced my research life, and one of the things I appreciated about V. Massey was that he liked to experiment. He was always experimenting in the gaps where the experimental machine was open. He was surprised when he learned that I loved experimenting as much as he did. We used to experiment on weekends and late at night. The ideas we obtained from our experiments were invaluable. This same love of experimentation was shared by Tomoko Nishino. Unfortunately, they reached retirement age and could no longer conduct experiments. Tomoko Nishino is a pediatric neurologist, and Takeshi Nishino is a professor emeritus at Nippon Medical School and a research supervisor at the University of Tokyo, Department of Agricultural and Life Science. Emile F. Pai and Russ Hill are my long-term contacts from roughly the same period; Emile Pai’s major contribution has already been mentioned in the above section on X-ray crystals. Russ Hille kindly invited me to join UCR after my retirement. Takeshi returned to Japan to live with his family.

**Figure 11:**
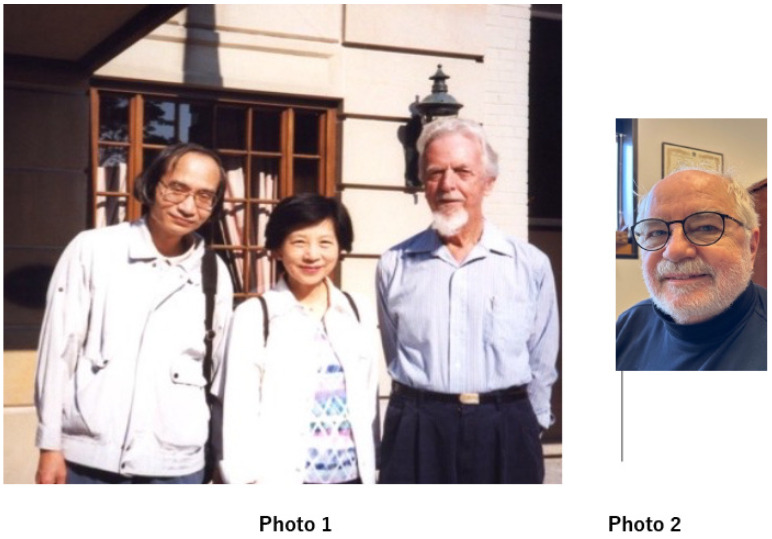
Photo 1: Takeshi Nishino (**left**), Tomoko Nishino (**center**), and Vincent Massey (**right**). Photo 2: Russ Hille.

## Data Availability

Not applicable.
